# Pathology and Practice of Medicine

**Published:** 1861-07

**Authors:** Richard J. Dunglison

**Affiliations:** One of the Physicians to the Pennsylvania Institution for the Instruction of the Blind; Honorary Local Secretary to the Sydenham Society of London, Etc.


### PATHOLOGY AND PRACTICE OF MEDICINE.

BY RICHARD J. DUNGLISON, M.D.,

ONE OF THE PHYSICIANS TO THE PENNSYLVANIA INSTITUTION FOR THE INSTRUCTION OF THE BLIND; HONORARY LOCAL SECRETARY TO THE SYDENHAM SOCIETY OF LONDON, ETC.

I.—*The Confounding of Persons as a Symptom of Insanity.* By DR. SNELL. (From the "Allgemeine Zeitschrift für Psychiatrie," Band xvii., ss. 545, 554. Translated for the "Medical Critic and Psychological Journal," London, April, 1861.)

Dr. Snell, Director of the Asylum at Hildesheim, has, for several years past, devoted much attention to the consideration of the confounding of persons as a symptom of insanity. It is most frequently met with in mania, (its absence here, indeed, being exceptional,) next in dementia following mania, and accompanied by excitement, not rarely in recent melancholia, and in all forms of melancholia accompanied by excitement, and in the various forms of monomania and apathetic dementia. It prevailed in more than half of the cases on admission into his asylum, and in only a third of those who have resided there for some time. It is more observable in proportion as the delirium is more general, and is of frequent occurrence in the delirious fever of acute diseases, as pneumonia and typhus.

Independently of illusions and hallucinations, the remarkable disposition which the insane have to confound objects seems to have a fundamental relation to a general psychological law, viz., the incapability of the insane to do other than move within the accustomed circle of thought; the patient, transporting his former perceptions direct to new objects, and confounding the one with the other, being far too much occupied with himself to adapt new ideas, derived from the outer world, to his old ideas. He is unable to comprehend his changed condition, either as regards the past or the future.

The paper terminates with the following conclusions:—

1. The confounding of persons, places, and objects is one of the most frequent symptoms of insanity. 2. It is one of the most certain and most easily detected symptoms of psychical disturbance. 3. It is proportionate to the degree and general character of the mental excitement, and is usually a favorable prognostic sign. 4. It is most frequently observed in cases of recent occurrence. 5. In the passage of the primary forms of psychoses into the so-called secondary forms, the confounding of persons not unfrequently disappears, and its doing so is here an unfavorable sign. 6. In the passage of the primary forms of mental disturbance into recovery, such disappearance is one of the most certain tests of recovery.

II.—*The Influence of Change of Posture on the Characters of Endocardial Murmurs; with an attempt to explain the Cause.* By SIDNEY RINGER, Esq., Medical Registrar to the Children's Hospital, London.

After a careful examination of a large number of mitral regurgitant, aortic obstructive and regurgitant murmurs, and one case of mitral obstruction, Mr.

Ringer found the following general proposition to hold good: Endocardial murmurs are louder, harsher, and lower pitched in the lying than in the sitting or standing posture. With the exception of one case of aortic obstructive disease, and one case of mitral regurgitation and aortic obstruction, this was found to hold universally good.

These variations can be caused either by some alteration in the valves or part causing the murmur, or by some alteration in the force of the blood, probably the latter. It is well known that the frequency of the pulsations of the heart is increased in the sitting over the lying, and the standing over the sitting posture. This increased frequency is accompanied with a diminution in the force of the heart's contractions. Moreover, the diminution in force is in proportion to the increased frequency.

Patients with visible and tortuous arteries were examined in the different postures, when, in every case, the visibility with each impulse of the heart was decidedly less in the standing than in the sitting or lying postures. It may be objected to this, that visible arteries occur mostly in persons suffering from aortic regurgitant disease, and that, as gravitation would act more strongly in the sitting than in the lying posture, more blood would flow back into the heart; consequently less would be propelled, and the visibility would be diminished. But the same diminution in the visibility of the artery occurred in patients who were free from aortic disease. Moreover, in those cases in which aortic regurgitation occurred, there was found to be a diminution of the visibility of the pulse in the standing over the sitting posture, in both of which postures the effects of gravitation are the same on the heart. It is thus probable that the rapidity of the circulation is much the same in all the different postures.

Having thus proved that the increased frequency and force of the heart are somewhat in inverse proportion, the author attempts to show the connection between the frequency of the heart's impulse and the alterations in the characters of endocardial murmurs above described.

1. In a case presenting both mitral regurgitant and aortic obstructive murmur, the pulse was the same in frequency in both the sitting and lying postures: the murmurs were found to be the same in both cases.

2. In cases in which the difference in both the pulse and murmurs were well marked, the murmurs were occasionally found unaffected by position. At these times the pulse was found to be equally unaffected by position.

3. On making those patients who had the difference in both murmurs and pulse well marked exert themselves, so that their pulse was unaffected by position, it was found that their murmurs were equally unaffected.

4. Patients, especially children, were occasionally nervous and frightened at the examination. In these both the pulse and murmurs were unaffected by change of posture; but, as their confidence became restored, the difference in the pulse manifested itself, and *pari passu* the murmurs also became affected by altered position.

5. In changing from one posture to the other, the alteration in frequency of the pulse gradually and not instantaneously manifested itself. So, likewise, the alteration in the murmurs became gradually manifest.

The murmurs accompanying femoral aneurisms were found to be similarly affected by a change of posture. The alteration in the intensity of cardiac murmurs by a change of posture is often very great; it appears to affect mitral murmurs more than aortic.

Mitral murmurs are not of the same intensity nor pitch at the commencement and termination of the systole. Thus they increase in intensity, and become heightened to their very termination. Can this increase be due to the rotation of the heart, by which its mitral orifice is brought more anteriorly? It is difficult to conceive this possible. However, it may be stated that, when the murmur is listened to posteriorly, the commencement of the murmur is most intense and highest pitched, the very reverse of what is heard in front; which would be expected, as the mitral orifice would be carried away from the posterior part of the spine.

May it be due to the heart being contracted, and consequently smaller, when the mitral orifice would be brought more anterior? or are both combined? If the alteration be due to the rotation of the heart, then tricuspid murmurs should be the reverse of mitral; namely, most intense at their commencement. But tricuspid murmurs are often so covered with pulmonary rhonchi, that it is extremely difficult to listen attentively to them.

The rest of the paper is devoted to the subject of the generation of murmurs, and to the description of experiments made to determine their causes.

III.—*Temporary Paralysis of the Ciliary Muscle of the Eye.* Four Cases; perfect Recovery. By George Lawson, Esq., F.R.C.S., Surgeon to the Great Northern Hospital, London. (Lancet, May 11, 1861, p. 458.)

The accommodation of the eye being performed through the agency of the ciliary muscle, any sudden loss of the power of adjusting the eye for near objects occurring in an eye which has always had normal vision, must depend on some impairment in the function of this muscle, which may be either partially or entirely paralyzed. From the few cases which have fallen under the observation of Mr. Lawson, this affection appears generally to come on either during a severe illness, or else, as is more frequently the case, is first discovered by the patient during the period of convalescence, when, on attempting to read, he finds that the lines appear misty, and that he is unable to distinguish the words.

The symptoms are those of a total or almost complete loss of the power of accommodation in the eye. The patient is unable to focus his eye for near objects, and surrounding bodies appear indistinct. In one of the cases the patient could see distinctly objects at a certain distance, could read at twenty-six feet the markings on the clock; in fact, she was able to appreciate those objects which were far enough off to allow the parallel rays proceeding from them to form a focus on her retina without any effort of adjustment of her own, while at the same time she could not clearly distinguish near objects or read large ordinary type. She could only make out with difficulty the letters of words in Canon type.

In each case, with a slightly convex glass, the patient was able to read clearly moderate-sized print at its focal distance. The lens rendered the object clear without magnifying it. The prognosis is, perhaps, favorable; for in the four cases cited a speedy recovery to good vision followed the treatment, which consisted in resting the eyes, (not allowing them to attempt reading,) the free use of iron, quinine, and purgatives, and cold sponging and splashing the body.

It is difficult to assign a true cause for this affection, or to suggest what pathological state produces it. It is a condition analogous to the partial or entire paralysis which frequently attacks certain muscles or sets of muscles, especially those of the lower extremity, thus allowing a preponderance of action to their opponents, and producing some of the forms of the non-congenital varus and valgus. Why certain muscles should, after a long and depressing illness, become paralyzed, while others in the same limb remain unaffected, it is difficult, if not impossible, to say; but the fact is well known, although we cannot comprehend in what manner such a result is effected.

IV.—*Case of Perforation of the Stomach occurring under unusual Circumstances.* By BENJAMIN BELL, F.R.C.S.E. (Edinburgh Medical Journal, March, 1861, p. 783.)

An interesting discussion was elicited in the Medico-Chirurgical Society of Edinburgh, on the 6th of February, 1861, by the history of a case of perforation of the stomach, presenting peculiar features, both in its history and the appearances observed after death. From a review of the whole case, Mr. Bell thinks it probable that the young man had been affected with chronic disease of the stomach for some weeks at least before his fatal illness. Such affections, we know, are often very insidious in their progress, not being invariably accompanied by such well-marked symptoms as the *post-mortem* appearances might seem to warrant.

The patient was not altogether well on the morning of an excursion he took with some companions the day before his death, and the exercise and food he indulged in on that day were very unfavorable to one in his circumstances. The exertion of walking and partly running five or six miles; the raw turnips which he ate; the successive drinks of cold water when heated; the beer he drank; the walk homeward soon after dinner, were all more or less calculated to prove hurtful to one who, there is reason to believe, was affected with chronic inflammation, or even ulceration of the stomach. On his return, exhausted and shivering, he was sent to bed; retching came on and recurred at intervals, the matter ejected from the stomach, at first colorless, soon changing to a dark-brown fluid resembling fæces, but not having a feculent odor; and the belly began to swell rapidly.

He had severe paroxysms of pain but no sickness or nausea—his vomiting seeming to arise from the quantity of wind which was collecting incessantly in his stomach. His pulse was feeble, his skin cold and livid. A mixture of brandy and laudanum given to him was vomited up again and again. A small dose of medicinal naphtha was retained, but the vomiting soon commenced anew, and the parietes of the whole abdomen appeared to be stretched to their utmost limits, the patient calling out several times that he was going to burst. A little emphysema was now detected in the right groin, among the loose cellular tissue near the femoral ring, and the progress toward death was very rapid. Death itself occurred almost suddenly.

Inspection twenty-two hours after death revealed the presence of two round openings, equaling in dimensions half a crown, about the middle of the posterior aspect of the stomach. Extensive emphysema existed in the cellular tissue surrounding the viscera of the abdomen, as well as interlobular emphysema of both lungs, in front. Mr. Bell is inclined to believe that perforation to a limited extent occurred at the time of his symptoms becoming all at once greatly and alarmingly aggravated with *tympanites*, although it is *possible* that it took place the day before, during his excursion, when he complained that he felt so suddenly weak and "done up." The writer is disposed to think the extensive perforation due to chemical action occurring after death, digestion being in progress, a large quantity of undigested food being discovered in the stomach.

### V.—*The Spread of Zymotic Diseases.*

1. *An Epidemic Tracked.* (Lancet, April, 1861.)
   *Recent Introduction of Yellow Fever into Port Royal, Jamaica.* By DR. BRYSON. (Ibid.)
2. *Quarantine and the Spread of Epidemic Diseases.* By GAVIN MILROY, M.D. (Medical Critic and Journal of Psychological Medicine, April, 1861.)
   *Outbreaks of Yellow Fever in Ships of War.* By GAVIN MILROY, M.D. (Lancet, April 20, p. 383.)
3. *Outbreak of Exanthematic Typhus at Liverpool.* (Medical Times and Gazette, April 13, p. 390.)
   *Imported Fever in Liverpool without Quarantine.* (Lancet, quoted in Dublin Medical Press, April 17, p. 270.)
   *The Egyptian Fever at Liverpool.* (Medical Times and Gazette, May 4, p. 473.)
   *The Fever at Liverpool.* (Dublin Medical Press, April 17, p. 273, and April 24, p. 286; Lancet, April 27, p. 424, and May 11, p. 472; Medical Times and Gazette, May 4, p. 477.)

1. In the words of the *Lancet*, there are few more important questions in connection with the management of the health of sailors in tropical climates, and the resolution of some difficult points alike interesting to science and to commerce, than the determination of the conditions under which epidemic diseases, such as yellow fever and cholera, arise and are diffused. It has been an old and with some is still a favorite belief, that yellow fever is a disease not infectious, but solely due to a peculiar constitution of the atmosphere, strictly limited, indeed, in locality and arbitrarily bounded in duration. In Dr. Bryson's valuable paper, presented to the Epidemiological Society, relating the successive outbursts of yellow fever in a consecutive series of her Majesty's ships, facts and conclusions were recently stated which lead almost irresistibly to the view that yellow fever is directly, intensely, and fatally infectious.

The ship Icarus had touched at Balize, in August. There the yellow fever raged. Soon after leaving, her crew were attacked with the disease. Out of 120 persons 37 died. When the vessel reached Port Royal, a pinnace was sent from the Imaum to assist in landing the crew. 11 out of 14 of the boat's crew from the Imaum were then also seized with yellow fever, and most of them died. Then one of the Imaum's crew, who had only come in contact with the officers of the Icarus and their baggage on shore, was taken with the disease, which spread indiscriminately through the ship, attacking 38 persons, old and young, of whom 17 died.

Again, the ship Hydra's boats were for two nights employed in rowing guard around the Icarus. Within two days the yellow fever attacked the crew of the Hydra. It spread through the ship, killing 8 of the crew; and was only extinguished by dispatching the Hydra to the northern latitude of Halifax, Nova Scotia.

Finally, when the Imaum became the seat of yellow fever, as above stated, after communication with the ill-fated Icarus, a number of supernumeraries were removed from her to the Barracouta, but as much as possible segregated from its crew. Nevertheless, it was soon evident that these men had brought the seeds of the fever with them. Cases of yellow fever occurred among the supernumeraries, and then it spread to the crew of the Barracouta, its fatal ravages being only checked by the ship running into temperate latitudes.

It is thus, concludes Dr. Bryson, as clear as evidence can well make it, that the crew of the Icarus brought the yellow fever into Port Royal, that they infected the boat's crew sent from the Imaum to remove the sick to the hospital; and that the Imaum's boat's crew next infected their shipmates, who had previously been in perfect health. In like manner the Icarus communicated the disease to the boat's crew sent from the Hydra, who also subsequently infected their shipmates. Next the crew of the Barracouta were infected by the supernumeraries sent from the Imaum.

Thus from one central focus, and within the space of a few weeks, was the yellow fever radiated in three distinct and distant directions, establishing at each point a fresh nidus of infection, capable of propagating itself to all susceptible persons coming within the sphere of its influence, and contributing another unequivocal and conclusive proof of the communicability of the disorder.

2. From yellow fever to quarantine the transition is easy, and we therefore pass at once to the subject of the value and uses of quaratine regulations, and to the importation of fever where such regulations are not in force. That quarantine has failed, and continues to fail, in effecting the object for which it was established, is patent to all, says Dr. Milroy, who makes the subject a matter of special study in one of the journals before us. The countries in which it is most systematically and stringently exercised are certainly not more free than others from pestilential visitations, and some of them, Portugal and Naples, for example, have suffered more frequently and severely of late years from destructive epidemics than Holland and Great Britain, where the ordinary restrictions of quarantine are comparatively in abeyance.

The writer next refers to the mode in which epidemic diseases arise and are propagated; the indispensable co-operation of an impure and vitiated atmosphere to the full force and malignity of all pestilential diseases; the various signs or phenomena which precede epidemics; the migratory character of such diseases, like the progression of insect swarms, from one region to another, and the history of the spread of yellow fever, cholera, and the "black death" from distant climes; and suggests that if accurate registers were kept of the exact dates of the development of the disease in different countries, and of other meteorological phenomena at the same time, we might some day make an approach to the discovery of a "law of epidemics."

The doctrine of "contagion" is next discussed, and the view that a disease must universally and invariably manifest a power of transmission from one person to another, because it has done so under certain special conditions, is not inaptly considered by the writer as untenable as that because trees may be multiplied by slips or cuttings planted in the ground, their propagation is never effected by their seeds being wafted from place to place. Dr. Milroy places the value of quarantine as an instrument of good, in removal of the sick into a healthy locality, away from the ship on board which they have sickened, not in the detention of the passengers and crew because they have recently arrived from sickly regions.

There is no reliable evidence, says the writer, to show that any pestilential disease was ever introduced into a country by the cargo of a ship, or by the ordinary articles of trade or merchandise; always provided that they were in a fresh, unputrescent condition, and excluding, of course, from the category such articles as the bedding and body clothing of the sick. Among the thousands of men who have been employed, since the beginning of the present century, in the lazarets of Malta, Marseilles, and Genoa, in handling the cotton bales and other goods on board vessels from Turkey and Egypt, not a single instance of sickness attributable to the occupation has occurred.

The general principles governing the proprieties of quarantine regulations thus laid down by Dr. Milroy seem to have been introductory to a further discussion of the same or kindred subjects. Thus we find in the *Lancet* some views by the same writer on "Outbreaks of Yellow Fever in Ships of War." An important suggestion is made which is not wholly applicable to Great Britain. It is this: Why should not a naval medical court-martial be held upon every vessel, which suffers loss or injury to human life, the evidence weighed, and the opinions of medical officers recorded, and important questions in naval hygiene settled? Could not the Epidemiological Society collect all scattered information upon outbreaks of the yellow fever pestilence on board ships, whether of war or of commerce, for the last ten years, or at least prepare a method of uniform observations for future cases?

3. Liverpool has lately afforded the Transatlantic medical journals a fertile field for the study of exanthematic typhus fever imported into that seaport. The facts are interesting. On the twenty-second of February, an Egyptian frigate, the Scheah Gehald, came into port from Alexandria, after a tedious voyage, with three hundred badly-clothed and half-starved people on board. About eighty of the crew were on the sick list, but no case of *fever* occurred during the voyage; nothing but dysentery or diarrhœa, a few chest affections, and three or four frost bites.

The condition of the men, however, exceeded in filth and stench anything that had ever come under the eyes and noses of the observers, and the lice on them constituted a phenomenon such as has not been witnessed since the age of the Pharaohs. Nevertheless, two hundred of them were taken, without quarantine or precaution of any kind, to a neighboring establishment of baths and wash-houses for ablution, and thirty-two to one of the general hospitals, in which they soon gave rise to fatal consequences. At the former establishment, the bathman, the keeper of the baths, and a female assistant were attacked with "typhus fever," while at the hospital the senior and junior house-surgeons, a student, a clergyman, two nurses, two porters, and nine patients became likewise affected. The character of the fever was that of true typhus.

It is important, says the *Medical Times*, to ascertain beyond doubt the following facts: 1. Whether really, as asserted, no single person among the Turkish crew had typhus, at the time when the disease was communicated to persons in Liverpool. 2. Whether the vessel during the voyage had had any cases of typhus fever, or whether any of the crew had had any communication with persons ill of that disease. 3. Whether it can be clearly made out that *all* the cases occurring at Liverpool among the inhabitants were really cases of exanthematic typhus. 4. Whether the disease seemed to be caused by emanations from the clothes or from the persons of the Turkish sailors who were dirty and sick, but who, it is stated, had not typhus.

Dr. Cameron, physician to the hospital, gives the following facts and views on the origin of the epidemic. The hygienic condition of the hospital after the admission of the Egyptians, and the prevalence of typhus fever in the locality in which the hospital is situated, might offer a satisfactory explanation; but a better may be found in the relationship that exists between typhus and dysentery, and, under certain circumstances, their probable convertibility the one into the other. Dr. Cameron thinks that the epidemics of fever which commit such havoc in our jails and emigrant ships are replaced, in the dark races, by a no less fatal dysentery.

If the general views of the writer be accepted as the correct explanation, we can comprehend how individuals in perfect health may suffer, when suddenly brought into contact with others who were saturated with the poisonous miasm, to the noxious influence of which they had, from gradual exposure, become insensible. In the discussion elicited upon this invasion of typhus fever in Liverpool, the ground is taken by some, and combated by others, that the darkness of the skin of the Egyptians prevented the surgeons from seeing the exanthematous manifestation of the disease.

In reviewing the history of these cases, the facts appear to be, that the specific poison of typhus was carried in the ship, without acting on persons who cannot be supposed to have been insusceptible: that when the sailors went to the hospital and baths, they carried along with them two conditions all-powerful in the production of disease: firstly, an effluvium arising from their filthy, unwholesome, and sickly bodies, which vitiated the surrounding air, and, secondly, clothes alive with animalculæ, and saturated, doubtless, with infectious matters; and that the contagion did not propagate itself beyond the persons first attacked.

It is rather a remarkable circumstance, that when the Egyptian sailors left Liverpool again, apparently in good health, in another vessel, fever broke out among them, and proved very fatal, both to the Europeans and to the Egyptians on board. If this be true, and this last disease was unmistakably typhus fever, the argument that the fever in the dark races assumes the form of dysentery would seem to lose some of its force.

The facts thus detailed are still undergoing investigation, and may therefore again demand our attention at a future time, should any new views be elicited.

VI.—*Progressive Locomotory Ataxy.* By M. DUCHENNE, of Boulogne. (London Lancet, January 19, 1861, p. 71.)

The Paris correspondent of the *Lancet* does not allow his English friends to be blind to the new theories and novel researches of the French pathologists. The *Parisian Medical Intelligence*, in the number quoted above, is mainly devoted to recent additions to the pathology of affections of the nervous system, and especially with reference to "that definite group of symptoms, identified and rescued from the chaos of nervous affections by this pathologist, (Duchenne,) and placed among the still obscure neuroses, under the title of 'Progressive Locomotory Ataxy.'"

The fundamental characteristics of this special disorder consist in the progressive diminution of the co-ordinating power, and in the simulation of paralysis, while muscular force continues in all its integrity, (as may be proved by the dynamometer.) The first morbid indication, often overlooked, is strabismus, the third pair of nerves or the sixth being implicated, and ocular divergence, or other deviation, accompanied by diplopia, is noticed. Its disappearance is followed by sudden attacks of acute lancinating pain occurring in different parts of the body, generally in the limbs, of an evanescent and neuralgic character, and, to the patient, resembling electric shocks.

These are followed by vertigo, vomiting, disturbance of the co-ordinating function, and increasing obtuseness of tactile sensibility, especially in the lower extremities—a train of symptoms which points more directly to the cerebral origin of the disease. The gait becomes uncertain and hurried, each movement of the leg resembling an awkward kick. The flexion of the various muscles of the limb in progression reminds one of the movements of a child's harlequin toy or marionette—a phenomenon referred by M. Duchenne in part to a disturbance of that harmony which, in the normal state, exists between muscles contracting under the stimulus of the will, and their special antagonists; in part to a loss of that instinctive or voluntary association inseparable from the working of the physical mechanism.

The patient loses the automatic portion of the co-ordinating function, and has to supply its place by the exercise of voluntary power. When all these symptoms increase in intensity, progression is rendered impossible, and, although no muscular paralysis exists, the patient is as thoroughly helpless as if stricken by paraplegia. Post-mortem examinations of the brain and spinal cord reveal no organic defect, and the experiments of M. Flourens serve to localize the disturbance in the cerebellum, the special organ of locomotive co-ordination. The treatment of this malady, in its various phases, has been hitherto completely unsuccessful.

VII.—*Statistics of Insanity in France.* By M. LEGOYT. Translated by J. W. BARSTOW, M.D., Resident Physician, Sanford Hall, L. I. (American Journal of Insanity, April, 1860, and April, 1861.)

In 1843, the French *Bureau de la Statisque Generale* published a series of tables, showing the condition and management of insane asylums in France, from 1835 to 1844, inclusive. In 1857, a continuation of the same series was published, giving similar statistics for the twelve years from 1842 to 1853, inclusive, and bringing the report forward to the close of 1853. The first part, relative to the eleven years, 1842–52, gives the general results of progress from year to year, with tables of admissions, discharges, etc.; the second part is devoted specially to the statistics of the year 1853.

Under the head of "Medical and Physiological Observations," it furnishes, as minutely as possible, a series of facts upon the development of insanity, upon the mortality and the results of treatment in this unfortunate class, by sex, age, civil condition, degree of education, occupation, etc.; also, some well-digested hints upon the probable causes of lunacy, making the usual triple distinction of the same into predisposing, physical, and moral.

There are two sources of error, however, which must detract somewhat from the value of the results, viz., the failure to classify the idiots and cretins in the various asylums, and the counting, among the admissions, of many cases which have been discharged, apparently well, and shortly readmitted, after a relapse; also, those who have made their escape from asylums, and are soon retaken; and, finally, those cases which are merely transferred from one asylum to another. Both these errors will be corrected in subsequent reports.

After alluding to the number of institutions in France devoted entirely or in part to the treatment of lunatics, (one hundred and eleven, including those of every class,) and to the population of all the asylums of France since 1835, M. Legoyt refers to the preponderance of female cases, owing to the fact that male patients invariably remain a shorter time under treatment than females, and also that the deaths of males in asylums greatly exceed in number those of females. The proportions are as follows:—

Males, in 1846, 48 to 100,000 inhabitants, or 1 in 2063.
In 1851, 57 to 100,000 inhabitants, or 1 in 1731.
Females, in 1846, 53 to 100,000 inhabitants, or 1 in 1877.
In 1851, 61 to 100,000 inhabitants, or 1 in 1625.

In looking at the *influence of socal crises* and agitations of the public mind upon the development of insanity, he considers that they have ceased to affect materially the public, simply by the fact of their frequent occurrence. The cholera of 1849 appears not to have exercised an influence corresponding to that of 1832, which, according to the testimony of many observers, had produced a large number of cases of mental disease, as the result of panic.

The relative *liability of the sexes* to mental disease next engages attention. The following table, according to M. Legoyt, seems to settle this question:—

| | Males. | Females. | Total. |
|---|---|---|---|
| Total number of admissions to French Asylums, (1842 to 1853,) . . . . | 50,194 | 43,975 | 94,169 |
| | Per cent. | Per cent. | Ratio of Males to Females. |
| | 53·30 | 46·70 | 1·14 to 1 |

He therefore confidently concludes that insanity is a disease to which men are more subject than women.

*Influence of Age.*—To sum up all the cases under treatment in 1853, and take the average age at which they are admitted to treatment, we find it:—

| | |
|---|---|
| For males . . . . . . . . . . . | 39 years 1 month. |
| " females . . . . . . . . . . | 41 years 9 months. |
| " both sexes . . . . . . . . . | 40 years 5 months. |

The mean age, therefore, of admission to French asylums is forty years and five months.

*Influence of Domestic Relations.*—One of the most remarkable facts brought to light by the tables collected, is the large proportion of unmarried persons admitted to the asylums, being for both sexes 61·80 per cent. of all those under treatment; while in the whole population at large, deducting all under fifteen years, there are only 36·74 per cent. unmarried inhabitants, being a proportion of more than one-third less than in the asylums.

*Professions and Vocations.*—Of 32,876 inmates of the various French asylums in 1853, it was found impossible to ascertain the professions of more than 27,620. Next to the soldiers and sailors, the liberal professions furnish the largest proportion of insane cases. After these, in order, come servants, domestics, day-laborers, etc., then mechanics, and finally those engaged in commercial pursuits. Looking at the class of liberal professions specially, we learn that the proportion of artists in the French asylums is nearly eight times greater than that of proprietors and tenants, seven times that of jurists, five times that of priests and physicians, and four times that of literary men.

The author then refers to the *causes of insanity* in nearly 20,000 cases, and regards hereditary predisposition as the chief predisposing cause; to the effect of seasons; the proportion of relapsed cases; the effect of city and country life; the nativity of those affected, etc.

Mental affections seem to be much more frequent among individuals born in those localities in the vicinity of the Seine, and also those which lie in the northwest of France, *i.e.* Bretagne and Normandy, while they are much less common among individuals born in the southern departments and the mountainous districts. Four of these departments, most remarkable for their industrial interests, stand at the head of those furnishing the greatest number of cases.

M. Legoyt then develops the necessity of prompt removal of patients from home upon the first manifestations of disease. During the prime of life the chances of recovery from mental derangement are most numerous, and these chances diminish rapidly as patients advance in age, especially as they approach the period when the mental powers naturally become enfeebled.

The average *age at which recoveries took place*, in 1853, is found to be as follows:—

| | |
|---|---|
| For males . . . . . . . . . . . | 36 years and 5 months. |
| " females . . . . . . . . . . | 38 years. |
| " both sexes . . . . . . . . | 37 years and 2 months. |

In the estate of marriage, the chances for recovery are greater than either in celibacy or widowhood, and in the widowed than in single persons; but we must remember that the class reported as single naturally includes all those whose insanity is dependent upon certain congenital or organic conditions which prevent the free development of intelligence, and which at the same time preclude marriage, as, for instance, idiots, cretins, etc.

Soldiers and sailors, and next those engaged in mercantile pursuits, present the largest number of recoveries. After these come mechanics; and the liberal professions rather oddly recover in just about the same proportion as laborers and servants.

The *relative mortality* among the insane in hospitals in France was six times greater than that of the population at large, owing to depressing causes which operate unfavorably upon the inmates of insane establishments, aside from the immediate influence of the mental disease.

The rest of this French contribution to statistical literature is devoted to local details, which, though eminently useful to a collector of minutiæ, would scarcely interest the American reader.*

* Those who desire to compare the statistics of insanity in France with similar results obtained from American sources will find all the facts at that time attainable in a paper entitled "Statistics of Insanity in the United States," by Richard J. Dunglison, M.D., published in this journal, in July, 1860.

